# COVID-19 in Multimorbid Patients—A Controlled Microcost Description Analysis of Diagnosis Related Group (DRG)-Case Series in Acute Care without Non-Invasive Ventilation

**DOI:** 10.3390/clinpract11040090

**Published:** 2021-10-08

**Authors:** Tobias Romeyke, Harald Stummer

**Affiliations:** 1 Institute for Management and Economics in Healthcare, UMIT Tirol—Private University for Health Sciences, Medical Informatics and Technology, 6060 Hall in Tirol, Austria; harald.stummer@umit.at; 2 Waldhausklinik, Acute Hospital for Internal Medicine, Pain Therapy, Complementary and Individualized Patient Centred Medicine, 86391 Deuringen, Germany

**Keywords:** COVID-19, costs, DRG, multimorbidity, LoS, micro-costing

## Abstract

Diagnosis-related cost analyzes are important for health economic planning and decision-making. They form the basis for further developing of remuneration systems for health services. The rapid increase in hospital stays by COVID-19 patients requires a valid and exact calculation of the treatment costs. COVID-19 patients with many accompanying illnesses increase the requirements for a cost calculation. The focus of this work is to carry out a DRG-related micro-cost analysis, considering the age, length of stay and comorbidities of COVID-19 patients. So far, there is little information about treatment costs for multimorbid patients with COVID-19 who have not received invasive ventilation. The method is based on a standardized cost unit calculation for determining the treatment costs in a German hospital. The costs (€) of inpatients treated with COVID-19 were compared with a control group of the same DRGs of patients without COVID-19. The average total costs for inpatient treatment were €2866. The highest share of costs falls on nursing, personnel, and material costs of the non-medical infrastructure. Frequent comorbidities were heart failure, diabetes mellitus, other respiratory diseases, dizziness, and impairment of the musculoskeletal system.

## 1. Introduction

Due to the rapid spread of the coronavirus disease 2019 (COVID-19) worldwide, it was declared a pandemic by the World Health Organization (WHO) at the beginning of March 2020 [1].

Many patients had to be treated as inpatients due to their state of health caused by COVID-19. Comorbidities, such as diabetes or high blood pressure, have a negative impact on the course of the disease, which often requires referral to intensive care units [2,3].

When the capacities of the ICUs (intensive care unit) become scarce, patients in smaller hospitals must continue to receive care. There are also patients who refuse machine treatment.

So far, there are few studies examining hospital costs caused by COVID-19. Some studies show global estimates of resource use in health care that may result from the pandemic [4]. It is also assumed that the medical costs caused by COVID-19 patients are higher than compared to other infectious diseases due to necessary hospital stays [4].

Although detailed, standardized cost records for inpatient COVID patients are still rare [5], the topic is of great international importance [6,7].

So far, several studies have been based on cost estimates [8,9] or simulation models [4].

A retrospective observational study deals with indirect and direct inpatient and outpatient costs of COVID patients, taking socio-demographic data into account [10].

Diagnosis-related cost analyzes are hardly available [11], and cost analyzes based on the system of diagnosis-related groups have not yet existed to our knowledge. DRGs (Diagnosis Related Group) combine many different combinations of diagnoses and procedures into groups with comparable economic costs and medically and clinically homogeneous groups. Each treatment case assigned to the same group receives the same financial resources. Various criteria, such as the main diagnosis, secondary diagnoses, the age of the patient, treatment procedures, ventilation hours and a few more, help with the allocation to the respective case group. DRGs enable budgeting and cost control and they influence strategic management decisions and form the basis for benchmarking. Cost analyzes are the basis for resource allocations [12].

The aim of this study is to determine costs for the care of COVID-19 patients according to the G-DRG system, taking concomitant diseases into account and using a standardized micro-cost-calculation method. Micro-costing is a direct listing of all costs that were required to treat an individual patient. It allows precise measurement of the costs of clinical interventions [13].

## 2. Methods

The application for the implementation of the empirical research project was approved by the Research Committee for Scientific Ethical Questions (UMIT—University for Health Sciences, Medical Informatics and Technology, Hall in Tirol, Austria) on 18 June 2021, under the reference number 2918.

The calculation of the treatment costs is based on the manual of the institute for the remuneration system in hospitals in Germany (InEK) [14]. The data was collected from 20 April to 20 December in an acute internal hospital in Germany (Waldhausklinik Deuringen, Stadtbergen, Germany) with an interdisciplinary focus. The costs for the comparison group correspond to the average costs for these DRGs incurred in German acute hospitals, based on InEK-data.

### 2.1. Microcost Analysis

The methodology section describes the collection of treatment costs, which are carried out in Germany using a standardized, validated procedure.

The individual costs are assigned directly to a cost unit (or a cost center). Overhead costs in a company summarize the costs that can only be allocated indirectly with the help of a distribution key and not directly to the individual cost units and cost centers (e.g., dressing materials and other consumables on the ward).

A distinction is made between “medical infrastructure” and “non-medical infrastructure” at the hospital that does not provide its services directly to the patient.

For the internal cost allocation procedure to be used, allocation keys are required that enable cost allocation based on the use of resources. In this study, performance statistics are used as the basis for a distribution of personnel costs. Indirect cost centers that do not provide their services directly to the patient are included in the cost center allocation.

Direct cost centers provide the services directly to the patient. In the context of cost unit accounting, the costs of the direct cost centers are allocated to the individual treatment cases. For the overhead costs of these cost centers, calculation rates are created for each service. The cost types posted to direct cost centers are made available after implementation the cost center allocation summarized in cost element groups (Table 1).

The case-related cost allocation takes place via a reference value calculation.

The calculation rates are based on suitable reference values formed, as in this study, the PPR minutes (Instrument for measuring the need for care in minutes) for the personnel costs of the nursing service.

A costing rate is calculated for each cost center that contains the determines the amount of costs incurred by a case when availing a service of the cost center is charged.

CR = C/WS

WS = CS1 × W1 + CS2 × W2 + … + CSn × Wn

CR = calculation rate of the direct cost center

C = calculation-relevant costs of the direct cost center (taking into account internal cost allocation)

WS = weighted services

CS1 = calculation-relevant services of type 1

CS2 = calculation-relevant services of type 2

CSn = calculation-relevant services of type n

W1 = weighting factor for services of type 1

W2 = weighting factor for services of type 2

Wn = weighting factor for services of type n

For the determination of the overhead costs to be allocated on a case-by-case basis.

Formula:

oc = CR × (nb1 × W1 + nb2 × W2 + … + nbn × Wn)

oc = overhead costs of the cost center that are assigned to the case

nb1 = number of benefits received during hospitalization of type 1

nb2 = number of benefits received during hospitalization of type 2

nbn = number of benefits received during hospitalization of type

This is a standard procedure in accordance with reference [14] [Inek (2016) based on the manual of the institute for the remuneration system in hospitals.

### 2.2. Complex Treatment of Colonization or Infection with Non-Multi-Resistant Pathogens Requiring Isolation (According to German Procedure Classification, OPS)

The OPS is an adaptation of the International Classification of Procedures in Medicine (ICPM) of the World Health Organisation (WHO). The patients suffering from COVID were treated as follows:

○ Treatment by specially trained medical staff, in cooperation with the hospital hygienist and/or the nurse for hospital hygiene (hygiene specialist) under the supervision of the hospital hygienist, considering current treatment and care standards
○ Carrying out special examinations to determine colonization or infection with a non-multi-resistant pathogen that requires isolation
○ Implementation of strict isolation (individual or cohort isolation) with your own sanitary area or bed chair (avoidance of cross infections). The isolation is maintained in accordance with the current guidelines of the Robert Koch Institute (RKI)
○ Change of bed linen, clothing, and personal care utensils (washcloths, etc.) in accordance with the current guidelines of the Robert Koch Institute (RKI), if necessary daily
○ Protective measures when entering and leaving the room (room-related protective gown, gloves, if necessary, mouth and nose protection, infiltration, outflow, etc.)
○ Special measures of hand disinfection before and after patient contact when dealing with spore-forming bacteria (alcoholic disinfection and washing of hands)
○ Daily disinfection of surfaces near the patient in accordance with the current guidelines of the Robert Koch Institute (RKI), if necessary, several times and/or using special surface disinfectants
○ At least daily floor disinfection and one-time final disinfection, if necessary, using special surface disinfectants
○ Discussions with patients and relatives (possibly also discussions with caregivers) on how to deal with non-multi-resistant pathogens that require isolation
○ Specific measures for the treatment or eradication of the pathogen according to the current recommendations of the RKI
○ Implementation of the following measures, if necessary:

Use of pathogen-specific chemotherapeutic agents/antibiotics

Implementation of diagnostic and therapeutic measures under special spatial and organizational conditions (e.g., in the patient room instead of in the functional area; if in functional areas, then with immediate final disinfection)

○ 8–98g.0 complex treatment on special isolation unit

Note:

○ 8–98g.00 up to 4 days of treatment
○ 8–98g.01 A minimum of 5 to a maximum of 9 treatment days

## 3. Results

The average age of the patients suffering from COVID-19 (SARS-CoV-2 reverse transcription polymerase chain reaction test was administered, following World Health Organization protocols (WHO 13.03.20)) was 74.4 years. The patients were treated in hospital for an average of 6.71 days. The OPS 8–98g was carried out. The treatment included a local oxygen supply, drug therapy for the main and concomitant diseases, as well as physiotherapy and physical measures.

Following DRG (Diagnosis Related Groups) cases with COVID-19 were examined:

⁻ DRG B85C (degenerative diseases of the nervous system)
⁻ DRG E79C (infections and inflammations of the respiratory organs)
⁻ DRG I68D (diseases and injuries of the spine not treated surgically)
⁻ DRG K60E (diabetes mellitus with severe comorbidities)
⁻ DRG K62C (various metabolic diseases)
⁻ DRG L60D (renal insufficiency)

**Figure 1:**
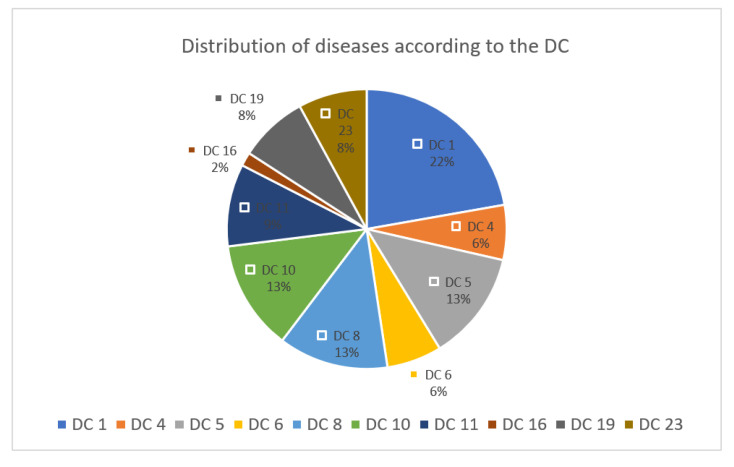
Distribution of secondary diagnoses from the examined COVID-19 patients. DC 1. Diseases and Disorders of the Nervous System. DC 4. Diseases and Disorders of the Respiratory System. DC 5. Diseases and Disorders of the Circulatory System. DC 6. Diseases and Disorders of the Digestive System. DC 8. Diseases and Disorders of the Musculoskeletal System And Connective Tissue. DC 10. Diseases and Disorders of the Endocrine, Nutritional And Metabolic System. DC 11. Diseases and Disorders of the Kidney And Urinary Tract. DC 16. Diseases and Disorders of the Blood and Blood Forming Organs and Immunological Disorders. DC 19. Mental Diseases and Disorders. DC 23. Factors Influencing Health Status and Other Contacts with Health Services.

We divided the secondary diagnoses into diagnosis categories (DC) (Figure 1).

A total of 73 diagnoses were made in the examined COVID-19 patients, 22% of them from DC 1, followed by DC 5, 8, 10 with 13% each. The treated COVID-19 patients had an average of 10.5 diagnoses. Frequent comorbidities were heart failure, diabetes mellitus, other respiratory diseases, dizziness, and impairment of the musculoskeletal system.

The costs (€) of inpatients treated with COVID-19 were compared with a control group of the same DRGs of patients without COVID-19.

**Figure 2:**
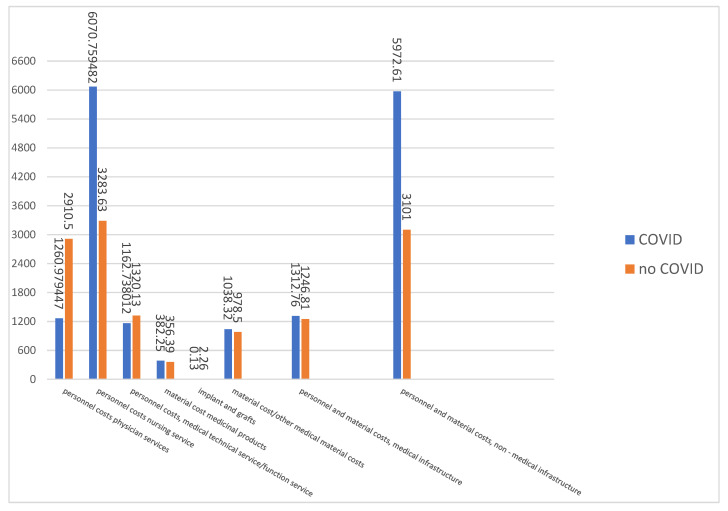
Comparison of total costs of inpatient treatment with and without COVID-19 (in €).

Nursing required to care for COVID-19 patients is almost twice as high as for conventional patients, while the medical effort is more than 60% lower. The highest share of costs falls on the personnel and material costs of the non-medical infrastructure. Compared to the care of conventional patients, the additional effort here is around 100%. For all other costs, it can be assumed that the costs for the care of COVID-19 patients and the control group are roughly the same for the DRGs examined (Figure 2).

## 4. Discussion

So far, there is little information about treatment costs for multimorbid patients with COVID-19 who have not received invasive ventilation.

A connection between comorbidities and hospital stays is assumed [15]. The multimorbidity criterion was met in all patients in this study [16].

It can be assumed that COVID-19 very often leads to an exacerbation of chronic diseases.

The results of this study show a dominant number of neurological comorbidities in COVID-19 patients. But cardiovascular and metabolic diseases and diseases of the musculoskeletal system are also frequently represented. This also coincides with the results of other studies [2,17].

The lying time partly coincides with the results of some other studies, whereby the lying time is generally to be regarded as rather short [18].

The average total costs for inpatient treatment were €2866. A hospital cost study conducted in China found a comparable average cost of $2869.4. This study by Dong et al. also refers to internet sources that provide cost information from other countries (USA, South Korea). Accordingly, the costs are higher in these countries [19].

Significant cost deviations between COVID-19 patients and the control patients in our study can be seen in the amount of care costs incurred and the personnal and material costs of non-medical infrastructure. This is since the care of a COVID-19 patient requires significantly higher staffing of nurses in all shifts. Due to the high expenditure in terms of the necessary hygiene measures, the use of materials, the insulation measures and the resulting high logistical effort in the hospital, the costs of cost element group 8 (personnel and non-material costs, medical infrastructure) are 100% higher. This also includes the PCR tests, the costs for house and cleaning staff, laundry, economic and utility services, energy, water, cleaning agents and disinfectants.

Cost calculations that are based on standardized process steps, create transparency for the health systems regarding the use of resources related to diagnosis [20].

Based on DRGs and the InEK calculation method, cost analyzes can be carried out for various clinical pictures [21].

Due to unexpected, special influences such as the COVID-19 pandemic, it is of great importance to collect and constantly update costs for inpatient hospital treatment. This allows adjustments to be made in the international DRG-based remuneration systems.

One of the strengths of the study is the micro-costing calculation method. This method ensures that the costs incurred for patient care are recorded precisely. It reflects the associated consumption of resources and the associated supply costs.

The effort involved in implementing this methodology is very high, but it allows the effort to be recorded with high precision [22]. A limitation of the study is that only a small number of DRGs were examined. The entire period of the pandemic was also not considered, only the year 2020.

## 5. Conclusions

The pandemic is putting the health systems worldwide under extreme pressure. In addition to the expansion of preventive protective measures against the disease, precise analyzes of the resource consumption in the international health systems must be initiated.

To increase the informative value of cost calculations for inpatient COVID-19 treatments, standardized cost unit accounting should be carried out, taking patient-related comorbidities into account. This includes all costs incurred for the individual patient, age, and length of stay. Micro-cost analyzes should also take the setting into account in addition to various DRGs, in particular for the care of patients in intensive care and normal wards.

These analyzes can make a valuable contribution to policy makers, managers, and stakeholders. Internationally, health expenditure is increasing every year. Therefore, the optimal use of limited health budgets is a priority for health policy. This fact also affects the managers of health facilities that they must maintain supplies with scarce resources, even in times of the pandemic.

In health economic analysis, standards for the implementation and publication of health economic studies have been given high priority. Stakeholders use these analyzes to identify trends on the one hand, maintain financial transparency and, on the other hand, to minimize risks.

## Figures and Tables

**Table 1:** Cost element groups (according to the InEK calculation manual [14]).

Cost Element Groups	Cost
Cost element group 1	personnel costs physician services
Cost element group 2	personnel costs nursing service
Cost element group 3	personnel costs, medical technical service/function service
Cost element group 4a,b	material cost medicinal products
Cost element group 5	implant and grafts
Cost element group 6a,b,c	material cost/other medical material costs
Cost element group 7	personnel and material costs, medical infrastructure
Cost element group 8	personnel and non-material costs, medical infrastructure

## Data Availability

The data of this article are not publicly available to protect personally identifiable information. And are available for requests of the corresponding authors.
